# Value of genetic testing in the prevention of coronary heart disease events

**DOI:** 10.1371/journal.pone.0210010

**Published:** 2019-01-15

**Authors:** Yrjänä Hynninen, Miika Linna, Eeva Vilkkumaa

**Affiliations:** 1 Department of Mathematics and Systems Analysis, School of Science, Aalto University, Espoo, Finland; 2 The Institute of Healthcare Engineering, Management, and Architecture, Department of Industrial Engineering and Management, School of Science, Aalto University, Espoo, Finland; 3 Department of Information and Service Management, School of Business, Aalto University, Helsinki, Finland; Massachusetts General Hospital, UNITED STATES

## Abstract

**Background:**

The health economic evidence about the value and optimal targeting of genetic testing in the prevention of coronary heart disease (CHD) events has remained limited and ambiguous. The objective of this study is to optimize the population-level use and targeting of genetic testing alongside traditional risk factors in the prevention of CHD events and, thereby, to assess the cost-benefit of genetic testing.

**Methods and findings:**

We compare several strategies for using traditional and genetic testing in the prevention of CHD through statin therapy. The targeting of tests to different patient segments within these strategies is optimized by using a decision-analytic model, in which a patient’s estimated risk of CHD is updated based on test results using Bayesian methods. We adopt the perspective of healthcare sector. The data for the model is exceptionally wide and combined from national healthcare registers, the Finnish Institute for Molecular Medicine, and published literature. Our results suggest that targeting genetic testing in an optimal way to those patients about which traditional risk factors do not provide sufficiently accurate information results in the highest expected net benefit. In particular, compared to the use of traditional risk factors only, the optimal use of genetic testing would decrease the expected costs of an average patient aged 45 years or more by 2.54€ in a 10-year follow-up period while maintaining the level of the expected health outcome. Thus, genetic testing is found to be a part of a cost-beneficial testing strategy alongside traditional risk factors. This conclusion is robust to reasonable changes in model inputs.

**Conclusions:**

If targeted optimally, the use of genetic testing alongside traditional risk factors is cost-beneficial in the prevention of CHD.

## Introduction

Coronary heart diseases (CHD) are the leading global cause of death, accounting for more than 7 million deaths per year [1]. Reducing the burden of CHDs and other cardiovascular diseases (CVD) with appropriate prevention is one of the objectives of the World Health Organization [2]. In order to target preventive interventions such as statin treatment in a cost-effective way, it is important to obtain reliable prognostic information on the patients’ state of health. Traditionally different risk measures based on clinical factors and lipid measurements, such as the Framingham Risk Score [3,4] or FINRISK function [5], have been used for this purpose. However, these measures are far from perfect: it has been estimated that more than half of all coronary heart disease events occur in individuals with estimated risk at low or average levels [6].

Over the last decade, research efforts have increased to discover the potential benefits of using genome information in the prevention of CVD [6–9]. Many studies have focused on one or a few candidate genes for CVD [10] and, more recently, genome-wide studies have tested tens of thousands of single-nucleotide polymorphisms (SNPs) in an effort to identify associations with CVD [7]. However, evidence—particularly health economic evidence—about the value of genetic testing has remained limited and ambiguous. On the one hand, it has been suggested that genetic testing strategies for cardiovascular diseases are more likely to be cost-effective than clinical tests alone [11,12] but, on the other hand, that the extent of cardiovascular disease risk reclassification would be small [7]. Specifically, economic evaluations of genetic testing have focused on intermediate outcomes such as cost per cases detected [13,14]. Studies which have explored the economic value of testing based on, e.g., cost-utility analysis are still relatively scarce [14]. A recent study evaluates the cost-effectiveness of a 27-SNP cardiovascular genetic risk score and concludes that genetic testing is generally not a cost-effective approach for targeting statin therapy for low- to intermediate-risk patients [15]. Yet, even in these more comprehensive analyses, the focus has been mainly on predetermined strategies in which, for instance, patient segments to be tested have been fixed in advance [15]. Additional challenges for reliable health economic analyses have been posed by inadequate data.

In this paper, we develop a decision-analytic model for optimizing the population-wide use of traditional risk factors and genetic testing in the prevention of CHD. We model a testing strategy as a decision tree in which the probability of a patient having CHD is updated based on test results using Bayesian methods. Based on optimal testing strategies, the cost-benefit of genetic testing compared to the use of traditional risk factors only can be reliably assessed. We utilize an extensive data set collected for the GeneRISK study [16] which includes, for instance, genetic test results of wide FINRISK cohorts and average CHD outcomes from national registries. We show that the use of genetic testing—if targeted optimally—is cost-beneficial in the prevention of CHD.

## Methods and materials

### Decision model

We developed a decision model to estimate the cost-benefit of population-based testing and treatment strategies for CHD. In this model, total costs and outcomes are measured from a healthcare sector perspective using a 10-year time horizon. We assume that the patient’s state of health is represented by a binary variable describing whether a patient will or will not have a CHD event in the following 10 years. This state of health is assumed to be static in that is does not change during the testing period. The length of the time horizon is aligned with the time horizons of the traditional risk score and genetic risk score. In the absence of additional information, prognosis about the patient’s state of health is made based on prior risk, i.e., the prior probability of the patient having a CHD event. The likelihood of a correct prognosis can be increased by carrying out tests on traditional risk factors, genetic risk factors, or both. Based on the prognosis, the patient is either treated with statin medication as primary prevention or not. In this paper, test results on traditional risk factors are represented by ‘Traditional Risk Score’ or ‘TRS’, which uses information about the patient’s sex, age, total cholesterol, high-density lipoprotein-cholesterol, systolic blood pressure, blood pressure treatment, smoking, prevalent diabetes, family history of myocardial infarction, and lipid treatment. The association between the factors of TRS and the rate of incident CHD events has been estimated by using a Cox proportional hazards model. As the ‘Genetic Risk Score’ or ‘GRS’ we use a novel score of 49,310 SNPs [17, and supplementary material thereof]. A detailed description of TRS and GRS is given in Appendix A of S1 File.

The decision model is used to compare six testing and treatment strategies:

(i) Do not test or treat any patient (‘No treatment’),
(ii) Use prior risk to determine who to treat with statin medication (‘Treatment optimized’),
(iii) Carry out TRS for an optimal patient segment to determine whether to treat or not (‘TRS optimized’),
(iv) Carry out GRS for an optimal patient segment to determine whether to treat or not (‘GRS optimized’),
(v) Carry out TRS for an optimal patient segment and, based on its results, optionally GRS for an optimal segment to determine whether to treat or not (‘TRS & GRS optimized’), and
(vi) Carry out GRS for an optimal patient segment and, based on its results, optionally TRS for an optimal segment to determine whether to treat or not (‘GRS & TRS optimized’).

**Fig 1:**
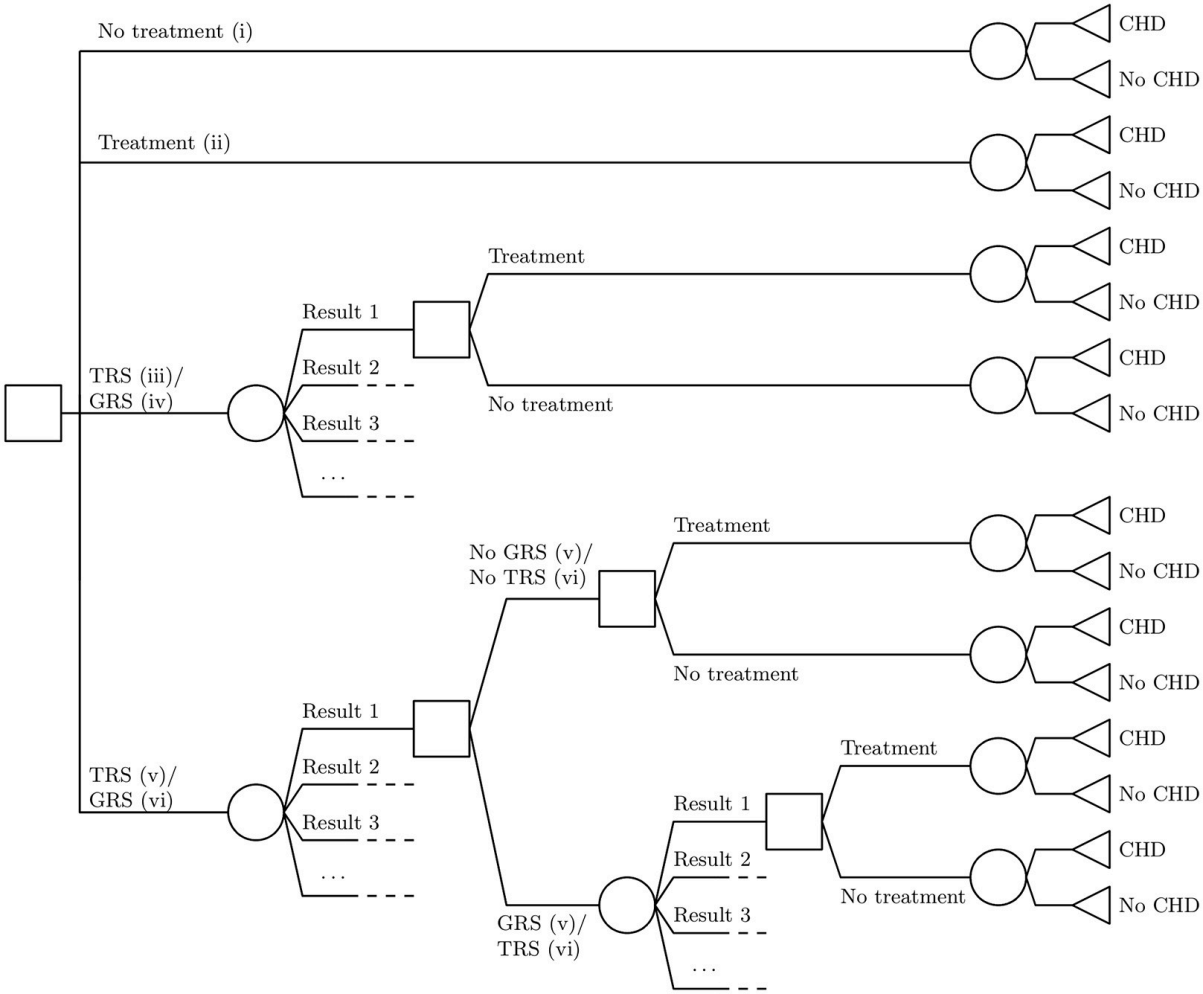
The decision tree of testing and treatment strategies (i)-(vi). TRS, Traditional Risk Score; GRS, Genetic Risk Score; CHD, coronary heart disease event.

The option to use strategies (i) and (ii) (‘No treatment’ and ‘Treatment optimized’) is embedded in strategies (iii)-(vi). The six testing and treatment strategies are presented as a decision tree in Fig 1.

In addition, we consider four reference strategies which represent predetermined, non-optimized testing strategies that might be applied in the absence of optimization models:

(vii) Carry out TRS for all patients (‘TRS for all’),
(viii) Carry out GRS for all patients (‘GRS for all’),
(ix) Carry out TRS for all patients and based on its results, carry out GRS for patients with updated risk between 10–20% (‘TRS for all & GRS for 10–20%’; adopted from the study of Tikkanen et al. (2013) [6]), and
(x) Carry out both TRS and GRS for all patients (‘TRS & GRS for all’).

Decisions about whether or not to test or treat are based on the patient’s estimate of risk, i.e., the probability of having a CHD event in the next ten years. A prior risk estimate is assumed to be known for every patient based on, for instance, studies in the literature (the average risk of a particular population) or known characteristics of the patient, such as gender and age. These prior risk estimates are updated based on observed test results using Bayesian methods [18].

The optimization of patient segments to be tested and treated at each stage is based on maximizing the expected net monetary benefit (NMB) as is typical in the approach of cost-benefit analysis [19]. Net monetary benefit is defined as

NMB=Healthoutcomes×λ−Costs,

where health outcomes are measured as quality-adjusted life-years (QALYs), costs as 2015 Euros, and λ by the societal willingness-to-pay (WTP) threshold (€/QALY). The approach of cost-benefit analysis was chosen because it enables the use of single-objective optimization methods.

To compute the expected NMB, we assume that both health outcomes and costs are known for each of the four possible outcomes of a testing and treatment strategy (cf. the end nodes in the decision tree in Fig 1):

I no CHD and no treatment (true negative),
II no CHD and treatment (false positive),
III CHD and no treatment (false negative), and
IV CHD and treatment (true positive).

In addition, health outcomes and costs associated with different tests are assumed to be known. The expected NMB of a strategy depends on the NMB of the above outcomes I-IV and the probabilities of these outcomes being realized, as well as the costs and health outcomes of testing.

Given a patient’s prior risk, the optimal testing and treatment strategy (i.e., the set of paths through the decision tree which maximize the expected NMB) can be solved by dynamic programming. Using a dynamic programming algorithm, the optimal decisions at the final stage are solved first (i.e., whether to treat or not) for all possible risk levels (0%, 1%,…,100%), after which the preceding decisions about whether or not to carry out GRS or TRS tests are solved recursively by taking into account the expected NMB resulting from subsequent decisions. The belief about the patient’s state of health is entirely contained in the risk estimate, meaning that the testing strategy through which this belief has been reached is irrelevant. The expected NMB corresponding to each strategy (i)-(vi) is determined by including only those branches of the decision tree in the computation that are included in the strategy.

To optimally segment patients for different clinical paths, the optimal strategies are solved for each prior risk (0%,1%,…,100%). As a result, the model gives the expected net monetary benefit (QALYs), the total costs (€), and health outcomes (QALYs) corresponding to the optimal strategy for each prior risk. The average expected NMB, total costs, and health outcomes are calculated as weighted averages of those corresponding to different prior risks such that the weights reflect the risk distribution in the population.

### Model inputs

#### CHD definition

In this study, a CHD event is defined as (i) hospitalization caused by unstable angina (I200; ICD-10), acute myocardial infarction (I21), subsequent ST elevation and non-ST elevation myocardial infarction (I22), or revascularization event, or (ii) death caused by diagnosis I20-I25, I46, R96, R98 (ICD-10). All following input data, i.e., the costs of a CHD event, the technical performance of TRS and GRS, and death incidence rates, are associated with this definition.

#### Risk distribution

The risk distribution used to determine the average expected NMB, costs, and health outcomes is calculated based on a population of 100,000 men and women aged 45 or more using the FINRISK function [5]. Here, the age distribution of the population is based on Finnish population in 2016 [20] and the health-related parameters (e.g., systolic blood pressure and cholesterol levels) are based on FINRISK 2012 research [21]. Details about the population and the FINRISK function are presented in Appendix B of S1 File.

#### Accuracy of testing

To update risk estimates based on observed test results using Bayesian methods, information on the accuracy of the tests is needed. Technically, this requires the assessment of the conditional probabilities of obtaining any test result given the patient’s state of health (CHD or no CHD). Here, the conditional probabilities are derived from the data of FINRISK studies with a total of 17,457 subjects (1992, 1997, 2002, and 2007 cohorts [22]), for which the results of TRS and GRS are known, as well as the observed 919 CHD events during the follow-up time of 10 years. We assume that the result of each test depends only on the patient’s state of health and not on the result of the other test, i.e., that TRS function and GRS are conditionally independent. This assumption is justified by the data which showed negligible correlation between traditional risk factors and GRS: the largest absolute value of correlation between TRS factors and GRS was 0.09.

#### Health outcomes

Estimates for the health outcomes of treatments and tests are based on literature reviews and national registers. Health-related quality-of-life (QoL) decrements, based on 15-dimensional (15D) [23] questionnaire estimates, were applied to each year spent in CHD-event states to calculate QALYs. As recommended by the US Panel on Cost-Effectiveness in Health and Medicine, a 3% annual discount rate was applied to both health outcomes and costs [24].

Table 1 presents the base case parameters and the corresponding distributions used in probabilistic sensitivity analysis. Table 2 shows the population-weighted averages of costs, health outcomes, and net monetary benefits of CHD events and treatments. Detailed data is presented in the following subsections.

**Table 1:** Base case values and distributions applied in probabilistic sensitivity analysis.

	Base case value	Probabilistic sensitivity analysis distribution	Base case source
**Health outcomes**			
CHD free (QoL)	0.90	Not varied	[20,25]
Disutility due to non-fatal CHD event (QALY)	-0.147	-1 × Beta(52,304)	[20,26–28]
Probability of death in case of event	22%	Beta(48,173)	[29]
Expected time of CHD event	5.75 years	Not varied	[5]
Risk reduction if statin treatment	-27%	-1 × Beta(45,121)	[30]
Annual side effect of statin treatment (QoL)	-0.002	Not varied	[31,32]
Discount rate of health outcomes	3%	Not varied	[24]
**Costs**			
Costs of obtaining traditional factors incl. blood panel, doctor and nurse visits (€)	173€	Not varied	[33]
Genetic testing (€)	200€	Unif(100,300)	Expert opinion
Annual statin costs (€/person)	53€	Not varied	[34,35]
Annual monitoring of a patient receiving statins (in primary prevention)	173€	Not varied	[33]
Annual secondary prevention	451€	Not varied	[36]
Non-fatal CHD event (undiscounted)	19,860€	Gamma(171,116)	National Discharge Register
Fatal CHD event (undiscounted)	2,417€	Gamma(171,14)	[35,36]
Willingness-to-pay threshold	50,000€		Assumption
Discount rate of costs	3%	Not varied	[24]

CHD, coronary heart disease; QoL, quality of life; QALY, quality-adjusted life-year.

**Table 2:** Costs, health outcomes and net health benefits.

	Treatment	No treatment
**Costs (€)**		
CHD event	-12,058	-14,629
No CHD event	-1,927	0
**Health outcomes (QALY)**		
CHD event	7.143	6.952
No CHD event	7.689	7.706
**Net monetary benefit (€)**		
CHD event	345,093	332,949
No CHD event	382,516	385,296

CHD, coronary heart disease; QALY quality-adjusted life-year.

Health outcomes of a CHD event: The QoL measures were obtained from surveys (15D) using a scale from 0 to 1, where 1 corresponds to “full health” and 0 to death. Table 3 presents the QoL estimates of CHD and CHD-free states. Here, the disutility due to a CHD event of patients aged 45–54 years is assumed to be the same as that of patients aged 55–64 years. The population-weighted average QoL of a subject in a CHD-free state is 0.90 [20,25], and the weighted average decrease in QoL resulting from non-fatal CHD events is -0.147 [20,26,27]. Assuming that the post-event QoL improves after one year to the level on which it was before the event, the health outcome of a non-fatal CHD event is -0.147 × 1 year = -0.147 QALYs. Based on the national cardiovascular disease register [29], the rate of death of patients with a CHD event is 22%. In case of death, the value of QoL was set at 0 between the expected time of the event and the end of the time horizon. Using the FINRISK function [5], the expected time of the event was found to be 5.75 years given that an event would occur within the 10-year time horizon.

**Table 3:** Quality of life estimates.

	Estimate (15D)	Source
**QOL of CHD-free state**		
45–54 years	0.935	[25]
55–64 years	0.920	[25]
65–74 years	0.900	[25]
75+ years	0.835	[25]
weighted average	0.903	[20]
**Disutility (QOL) due to CHD event**		
45–54 years	-0.177	[26,27], assumption
55–64 years	-0.177	[26,27]
65–74 years	-0.144	[26,27]
75–84 years	-0.085	[26,27]
85+ years	-0.012	[26,27]
weighted average	-0.147	[20]

QOL, quality of life; CHD, coronary heart disease.

Impact of statin medication on health outcomes: Based on a large meta-analysis [30], statins were estimated to decrease a patient’s risk for a fatal or non-fatal CHD event by 27%. In our model, we assume full compliance with statin medication, and apply a small quality-of-life decrement of 0.002 for each year spent receiving statin therapy [31,32].

#### Costs

Cost estimates of CHD events, treatments, and tests from the perspective of the healthcare sector were derived from national registers and literature. These estimates were adjusted to 2015 level using the healthcare price index published by the Association of Finnish Local and Regional Authorities [37], and then transformed into QALYs using a WTP threshold value. A 3% annual discount rate was applied [24]. Base case data on costs is presented in Table 1 and detailed data below.

Costs of testing: The cost of carrying out TRS was estimated to be 173€, including doctor and nurse visits and a blood panel, the prices of which were based on Finnish standard healthcare costs [33]. In our base case, the cost of GRS was assumed to be 200€ based on discussions with subject experts. We did not assume any fixed costs related to separate testing stages.

Costs of statin medication: Based on the distribution of different statins among new Finnish statin users starting treatment in 9/2007-12/2007 and reference prices for these statins in April 2013, the average annual medication cost of treating dyslipidemia was estimated to be around 53€ for both men and women [34,35]. The monitoring cost for a patient using statins was estimated to be 173€, based on the assumption that monitoring requires the same visits and tests as carrying out the original TRS, i.e., one additional doctor, nurse, and laboratory visit annually, priced according to Finnish standard healthcare costs [33]. In our model, we assumed that medication is constant for the examination period of 10 years. In case of a fatal CHD event, statin medication stops at the expected time of the event (5.75 years).

Costs of a CHD event: The costs of different non-fatal CHD events (myocardial infarction, unstable angina pectoris, and revascularization) include the average cost of treating an acute event in special healthcare and one-year follow-up in both primary and special healthcare. Table 4 presents the costs and frequencies of CHD events. The weighted averages are used to estimate the mean cost of a non-fatal CHD event: 9,015€ + 10,844€ = 19,860€. In our model, we assume that secondary prevention is carried out for every survived patient beginning at the expected time of a non-fatal CHD event (5.75 years) until the end of the time horizon. Based on the same monitoring costs as with statin treatment and the yearly costs of medication used in the secondary prevention of coronary heart disease according to Finnish Current Care Guidelines [38], the average annual cost for a patient in secondary prevention was estimated to be 451€. The cost of a fatal CHD event was assumed to be 2,417€ [35,36].

**Table 4:** Costs of CHD events in 2011 (in 2015 value).

	Acute event	1-year follow-up	Number of events	Source
MI	8,585€	9,814€	11,406	National Discharge Register
AP (unstable)	6,702€	10,147€	2,689	National Discharge Register
CABG	19,483€	23,666€	2,493	National Discharge Register
PTCA	7,122€	8,523€	7,901	National Discharge Register
Weighted average	9,015€	10,844€		

CHD, coronary heart disease; MI, myocardial infarction; AP, angina pectoris; CABG, coronary artery bypass grafting; PTCA, percutaneous transluminal coronary angioplasty.

## Results

### Base case analysis

The optimal segmentation of patients for different clinical paths as well as the optimal testing and treatment decisions for these segments were solved for each strategy (i)-(vi). Based on this information, the expected net monetary benefit as well as the expected total costs and health outcomes were calculated for optimized strategies (i)-(vi) and non-optimized reference strategies (vii)-(x). Table 5 presents the expected net monetary benefits, total costs, and health outcomes per average patient corresponding to each strategy. The strategies are sorted in decreasing order of their expected NMB.

**Table 5:** The expected net monetary benefits, costs, and health outcomes of the strategies.

Strategies	Net monetary benefit (€)	Cost (€)	Health outcome (QALY)
(v) TRS & GRS optimized	379,786	1,674	7.6292
(iii) TRS optimized	379,784	1,676	7.6292
(vi) GRS & TRS optimized	379,733	1,707	7.6288
(ix) TRS for all & GRS for 10–20%	379,710	1,800	7.6302
(vii) TRS for all	379,709	1,801	7.6302
(iv) GRS optimized	379,625	1,700	7.6265
(ii) Treatment optimized	379,613	1,697	7.6262
(x) TRS & GRS for all	379,537	1,988	7.6305
(viii) GRS for all	379,444	1,891	7.6267
(i) No treatment	379,159	1,646	7.6161

TRS, traditional risk score; GRS, genetic risk score.

Strategy ‘TRS & GRS optimized’, in which GRS is carried out optionally after having observed the result of optimally targeted TRS, has the highest NMB of 379,786 € = 7.6292 QALYs × 50,000 €/QALY– 1,674 € and is therefore optimal. The second-highest NMB = 379,784 € is obtained with strategy ‘TRS optimized’, in which TRS is carried out to an optimal patient segment without additional genetic testing. This strategy yields approximately the same expected health outcome as ‘TRS & GRS optimized’ but has a higher expected cost, whereby strategy ‘TRS optimized’ is dominated by strategy ‘TRS & GRS optimized’. The remaining strategies have essentially lower NMBs than ‘TRS & GRS optimized’, because they would result in either (i) a substantial increase in costs relative to the increase in health outcomes (strategies ‘TRS for all & GRS for 10–20%’, ‘TRS for all’, and ‘TRS & GRS for all’), (ii) a substantial decrease in health outcomes relative to the decrease in costs (strategy ‘No treatment’), or (iii) a decrease in health outcomes and an increase in costs (dominated strategies ‘GRS & TRS optimized’, ‘GRS optimized’, ‘Treatment optimized’, and ‘GRS for all’).

**Fig 2:**
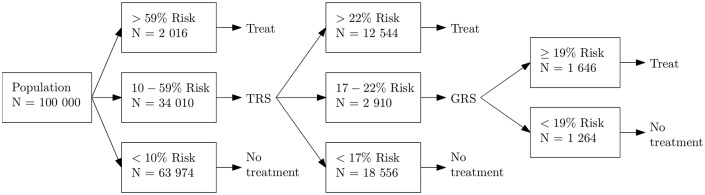
Base case optimal strategy in a population of 100,000 people. TRS, Traditional Risk Score; GRS, Genetic Risk Score.

The optimal decisions corresponding to the optimal strategy ‘TRS & GRS optimized’ are illustrated in Fig 2 in which the expected number of patients in each segment corresponds to a Finnish population of 100,000 patients aged 45 or more. Approximately 34% of the population is expected to be tested with TRS and 3% with GRS. In total, (2,016 + 12,544 + 1,646)/100,000 = 16% of the population is expected to be treated. Compared to strategy ‘TRS optimized’, in which the optimal segment to be tested with TRS is the same (10–59%), the optimal inclusion of genetic testing changes the testing strategy of 2,910 patients whose updated risk after TRS is 17–22% (see Fig 2).

### Sensitivity analyses

To investigate the sensitivity of our results to the model parameters, we conducted a probabilistic sensitivity analysis (PSA), a one-way sensitivity analysis, and a two-way sensitivity analysis. In the PSA, we sampled combinations of parameter values from the distributions specified in Table 1. These distributions were selected such that (i) the expected value of each parameter would coincide with its base case value, and (ii) the 95% confidence interval for this value would be

- The base case value ±15% for the cost of non-fatal and fatal CHD event,
- The base case value ±25% for the disutility due to non-fatal event, probability of death in case of event, and risk reduction if statin treatment, and
- The base case value ±50% for the cost of genetic testing.

Using these distributions, we carried out 1,000 Monte Carlo trials for each value of the WTP threshold 0€, 2,000€, 4,000€,…, 98,000€, 100,000€. In each trial, the segmentation of patients and the corresponding testing and treatment decisions were optimized for each strategy (i)-(vi) given the sampled parameter values and the WTP threshold. Then, the expected NMBs were computed for all strategies (i)-(x), based on which we could determine whether strategy ‘TRS & GRS optimized’ would be optimal, i.e., if it would yield the highest expected NMB among strategies (i)-(x).

**Fig 3:**
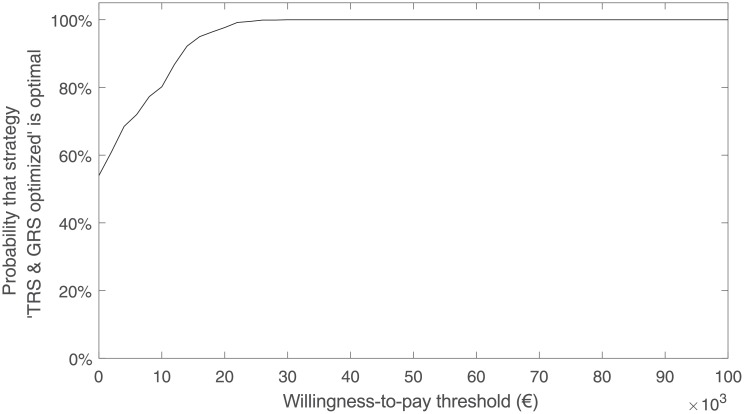
Probabilistic sensitivity analysis of strategy ‘TRS & GRS optimized’.

The results of the PSA are illustrated in Fig 3 through a curve that describes the share of Monte Carlo trials in which strategy ‘TRS & GRS optimized’ was optimal for different values of the WTP threshold. Assuming that the parameter values are independent of one another, this share can be interpreted as the probability with which strategy ‘TRS & GRS optimized’ is optimal. For instance, for a WTP threshold of 10,000€/QALY, the probability of strategy ‘TRS & GRS optimized’ being optimal is 80%, whereas for a WTP threshold of 20,000€/QALY this probability is 98%. For all WTP threshold values over 26,000€/QALY, strategy ‘TRS & GRS optimized’ is optimal with 100% probability.

**Fig 4:**
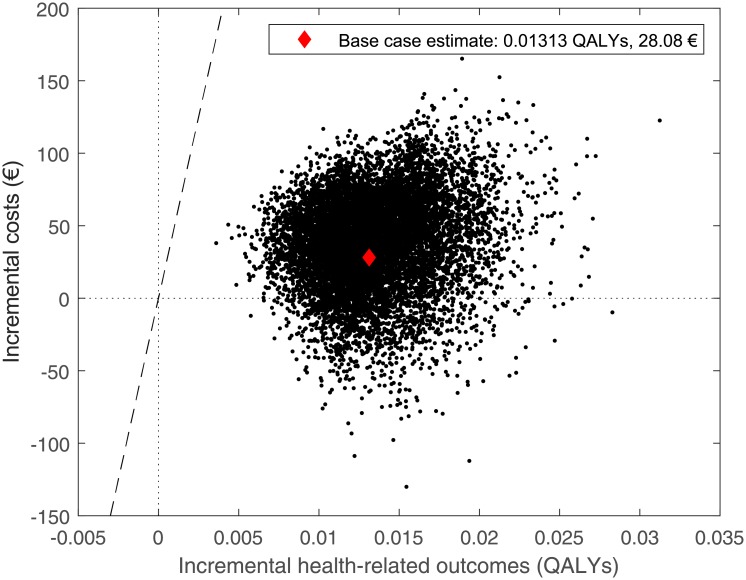
Scatter plot depicting the incremental health-related outcomes and costs of strategy ‘TRS & GRS optimized’ versus strategy ‘No treatment’ derived from the Monte Carlo (probabilistic) sensitivity analysis. Each dot represents the incremental health-related outcomes and costs of one sample (i.e., the population-weighted averages). Strategy ‘TRS & GRS optimized’ was optimized for each set of sampled parameter values using the willingness-to-pay threshold of 50,000 €/QALY.

If strategy ‘TRS & GRS optimized’ is optimal in a given trial, this means that the other strategies (i) are dominated (i.e., have both lower expected health outcome and higher expected cost), (ii) increase both the expected cost and health outcome such that the cost increase relative to the increase in health outcome is larger than the WTP threshold, or (iii) decrease both the expected cost and health outcome such that the cost decrease relative to the decrease in health outcome is smaller than the WTP threshold. In such trials, strategy ‘TRS & GRS optimized’ can be seen as cost-effective in the sense that no other strategy would increase the expected health outcome with an incremental cost-effectiveness ratio (ICER) lower than the given WTP threshold. In fact, Fig 3 can be compared to the cost-effectiveness acceptability curve (CEAC), which is a standard way of presenting PSA results of cost-effectiveness analyses. Whereas CEAC presents the probability with which a predetermined, non-optimized strategy has an ICER below some specific value when compared to a given reference strategy, Fig 3 presents the probability with which an optimized strategy has the highest expected NMB among several (optimized or non-optimized) strategies for a specific WTP threshold value. Scatter plot from the PSA comparing strategies ‘TRS & GRS optimized’ and ‘No treatment’ is presented in Fig 4.

In addition to the PSA, we conducted several one-way sensitivity analyses of the net monetary benefit of strategy ‘TRS & GRS optimized’ by varying the following parameters: costs of fatal and non-fatal CHD events (±15%), cost of TRS (±25%), cost of GRS (±50%), cost of treatment (±25%), and health outcomes of a CVD event and a treatment (±25%). Again, the segmentation of patients and the corresponding testing and treatment decisions were optimized for each set of different parameter values. The results of these one-way sensitivity analyses are presented in Table 6. According to the results, the net monetary benefit of strategy ‘TRS & GRS optimized’ is most sensitive to changes in (i) the probability of death in case of a CHD event, (ii) risk reduction by statins, and (iii) the cost of a non-fatal CHD event. However, despite the changes in net monetary benefit resulting from parameter variation, strategy ‘TRS & GRS optimized’ remains the optimal strategy with the highest net monetary benefit compared to other strategies.

**Table 6:** One-way sensitivity analysis of the net monetary benefit of strategy ‘TRS & GRS optimized’.

	Net monetary benefit (€)
***Base case***	***379 786€***
**Cost of a fatal CHD event** **(base case 2 417€; ±15%)**	
2 055€	+6€
2 780€	-6€
**Cost of a non-fatal CHD event** **(base case 19 860€; ±15%)**	
16 881€	+180€
22 839€	-179€
**Annual statin costs** **(base case 53€; ±25%)**	
40€	+16€
66€	-15€
**Annual monitoring costs in primary prevention** **(base case 173€; ±25%)**	
130€	+56€
217€	-49€
**Annual secondary prevention costs** **(base case: 451€; ±25%)**	
339€	+27€
564€	-27€
**Disutility due to a non-fatal CHD event** **(base case -0.147 QALY; ±25%)**	
-0.110 QALY	+111€
-0.184 QALY	-111€
**Probability of death in case of a CHD event** **(base case 22%; ±25%)**	
16%	+639€
27%	-629€
**Risk reduction if statin treatment** **(base case 27%; ±25%)**	
20%	-254€
34%	+281€
**Cost of TRS** **(base case 173€; ±25%)**	
130€	+18€
217€	-15€
**Cost of GRS** **(base case 200€; ±50%)**	
100€	+5€
300€	-2€

**Fig 5:**
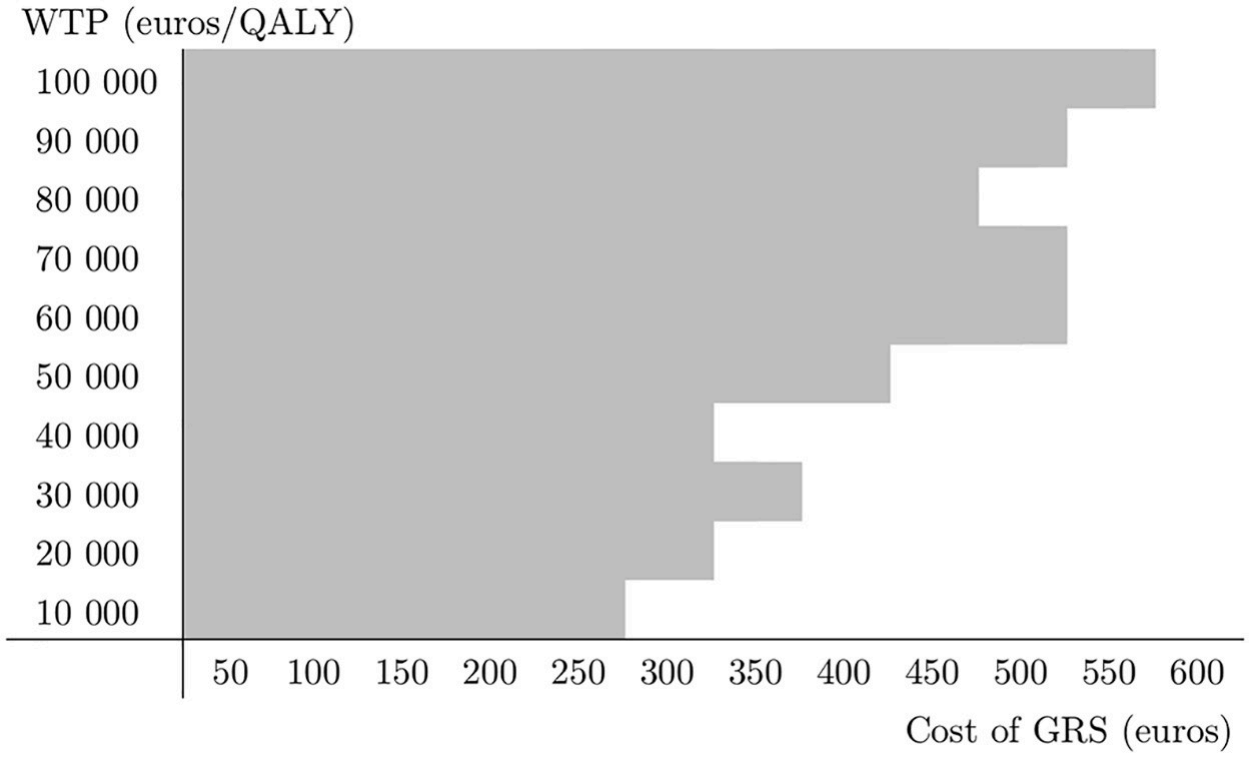
Two-way sensitivity analysis of WTP threshold and the cost of GRS. The gray area represents the WTP-cost combinations for which strategy ‘TRS & GRS optimized’ yields the highest net monetary benefit, i.e., for which GRS is a part of the optimal testing strategy. WTP, willingness-to-pay; QALY, quality-adjusted life-year; GRS, Genetic Risk Score.

Finally, we conducted a two-way sensitivity analysis on the WTP threshold and the cost of GRS. The WTP threshold is a highly contested value [39], which influences many other factors in the computation. On the other hand, the cost of GRS is an interesting variable because it influences decision making essentially and has decreased substantially in the last few years. The results of the two-way sensitivity analysis are visualized in Fig 5, where the gray area corresponds to those parameter combinations of the WTP threshold and the cost of GRS with which strategy ‘TRS & GRS optimized’ is optimal. This Figure suggests that if the price of GRS is 250€ or less, strategy ‘TRS & GRS optimized’ is optimal regardless of the WTP threshold. On the other hand, if the WTP threshold is 50,000€ as in the base case, then strategy ‘TRS & GRS optimized’ is optimal whenever GRS costs less than 450€.

## Discussion

In this paper we built a decision-analytic model to optimize the use of traditional and genetic testing to support the targeting of statin medication treatment in preventing coronary heart disease. Using the model, we were able to assess the cost-benefit of genetic testing from the perspective of healthcare sector. Our results suggest that genetic testing is a promisingly cost-effective technology in the prevention of CHD: Compared to the optimal use of traditional risk factors only, the inclusion of genetic testing would decrease the expected costs of a single patient by 2.54€ during a 10-year follow-up period while maintaining approximately the level of the expected health outcome. In a Finnish population of 100,000 patients aged 45 or more, genetic testing would be expected to be carried out for 2,910 patients, decreasing the total costs by 254,000€ and increasing the health outcome by 1.7 QALYs.

Our work has some limitations. First, our model is static in that it considers the risk of a patient having a CHD event in the following ten years without explicitly modeling the deterioration in the patients’ health over time. In this respect, combining our decision-analytic model with a Markov model would increase the relevance of our results by supporting the optimal timing of interventions as well. Second, our model did not account for information provided by genetic testing about other diseases besides CHD, or about the state of health of the testees’ family members who share the same genetic traits. In addition, we did not account for the impact that the acquisition of genetic risk information might have on the patients’ adherence or motivation to improve their lifestyle habits. Third, we considered statin therapy as the only treatment option, although other options for mitigating the risk for CHD are available, such as health coaching interventions (albeit data about their effectiveness might be difficult to obtain). In particular, patients with a high risk of CHD tend to be on multiple drugs alongside statin, including angiotensin-converting enzyme inhibitors for treating hypertension. Ideally, the model should be extended to accommodate the costs and health-related impacts of all relevant interventions. Yet, in the absence of such data, preliminary conclusions about the use of multiple drugs can be drawn from sensitivity analyses on the cost of medication. Specifically, if the cost of medication increases due to the use of multiple drugs, then a smaller segment of patients will be treated, whereby the importance of a correct targeting of treatment is emphasized even more. Consequently, the incremental net benefit of being able to improve prognostic accuracy through genetic testing increases. For instance, when the annual cost of medication and monitoring is doubled from 226€ to 452€, then the optimal share of patients tested with GRS increases from 3% to 4%. Fourth, the GRS used in this study has recently been superseded by multiple groups [40,41], whereby the overall benefits of genetic testing in the optimal targeting of preventive treatment options may have been crudely underestimated. Finally, the price of genome sequencing continues to decline, which further increases its potential usefulness in the prevention and treatment of not only CHD but other chronic diseases as well.

To our knowledge, this paper is the first to optimize the targeting of genetic tests to different patient segments in the prevention of CHD. As illustrated by the comparison between optimized and predetermined, non-optimized testing strategies, optimization is crucially important in assessing the cost-benefit of testing technologies: The three strategies with the highest expected net monetary benefits were all optimized strategies, whereas the four non-optimized strategies were clearly the most expensive but did not result in a substantial increase in health outcomes. Consequently, to obtain justifiable results about the cost-benefit of new tests in the prevention of CHD or other diseases, it is necessary to optimize the entire population-level testing strategy instead of, e.g., following the conventional approach of targeting these new tests to some predetermined risk categories. If the required data can be obtained, the model presented in this paper can be readily applied to optimize the use of genetic testing (or other prognostic/diagnostic innovations) in the context of any disease.

The contribution of this study is further strengthened by the access to extensive patient data obtained from the Finnish Institute for Molecular Medicine and national healthcare registers. This data made it possible to estimate the performance of traditional and genetic risk scores as well as the outcomes and costs of CHD events. Combining information from various sources is indeed a prerequisite for obtaining reliable assessments about the cost-effectiveness of different measures taken to prevent CHD or other diseases. For this purpose, among others, the Finnish government decided in 2016 to invest 17 million euros in a new national center which integrates all genome, biobank, and healthcare utilization registers [42]. Data collected by this center would enable the acquisition of more accurate risk estimates for individual patients which, in turn, would result in more accurate cost-effectiveness analyses about new tests. The development of predictive models that can be used to map these data into patients’ risk estimates provides an interesting topic for future work.

## Supporting information

S1 File Appendices. (PDF)
