# Supplementary material for: Value of genetic testing in the prevention of coronary heart disease events
Source: PLoS One. 2019 Jan 15;14(1):e0210010. doi: 10.1371/journal.pone.0210010 (PMC6333335; doi:10.1371/journal.pone.0210010)
Supplement: S1 File — (PDF) [file pone.0210010.s001.pdf]

Appendix A – Risk scores

The association between the factors of the Traditional Risk Score (TRS) and the rate of incident CHD events has been estimated by using a Cox proportional hazards model on the following traditional risk factors: sex, log(total cholesterol), log(high-density lipoprotein-cholesterol), log(systolic blood pressure), blood pressure treatment, smoking, prevalent diabetes, family history of myocardial infarction, and lipid treatment

The Cox model is adjusted for the traditional risk factors at baseline and uses age as the time scale. Hazard rate is assumed to be constant over time, whereby the TRS is computed by using an exponential survival function. Similar risk scores have been used in the studies of Ripatti et al. (2010) [1] and Tikkanen et al. (2013) [2].

Conventional risk models such as the one used in this study or FINRISK function [3] or Framingham risk score [4,5] can be web-based or integrated to patient information system. The information obtained from them is a single number, i.e., a risk score, which represents the probability of having a CHD event in the following 10 years. Based on this risk score, there can be interpretation guidelines and intervention policies in the healthcare system.

As the genetic risk score (GRS), we use a score of 49,310 SNPs (GRS49K) [6]. For detailed information about GRS49K, see Abraham et al. (2016) and supplementary material thereof [6]. GRS49K also returns a single risk score for each tested patient.

Appendix B – Population distribution

We use FINRISK function [3] to calculate the prior risk distribution of Finnish population of 100,000 men and women aged 45 years or more [7]. All parameters needed in this function are obtained from the Finnish FINRISK 2012 study [8] and are presented in Table A. Our base case is a patient with no personal or family history of MI or stroke, no diabetes, not smoking. The 10-year-risk of CHD (i.e., the probability of having a CHD event in the next 10 years) is calculated separately for men and women in each age group using age-specific parameter values, after which the prior risk distribution of the population is obtained by summing the number of men and women with the particular CHD risk. The distribution is presented in Fig A.

Table A. Parameters for calculating 10-year risk estimates [8].

	Men	Women
**Cholesterol (mmol/L)**		
45-54 years	5.65	5.47
55-64 years	5.37	5.78
65-74 years	4.99	5.64
**HDL Cholesterol (mmol/L)**		
45-54 years	1.38	1.64
55-64 years	1.34	1.65
65-74 years	1.32	1.64
**Systolic blood pressure (mmHg)**		
45-54 years	134.0	130.4
55-64 years	140.8	137.1
65-74 years	143.8	145.6

HDL, high-density lipoprotein.

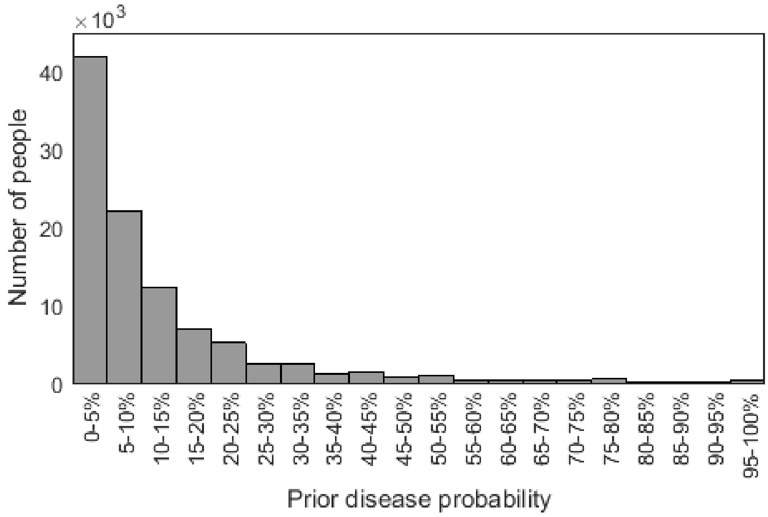

Fig A. CHD event risk distribution of 100,000 Finnish people aged 45 years or more.

Expected timing of CHD event

Consider survival function $S(t)$ which captures the probability that a patient will survive beyond a specific age t. The 10-year risk estimate $R_{10}(t)$, i.e., the probability at age t of having a CHD event in the next ten years, can be written as

$$R_{10}(t) = 1 - \frac{S(t+10)}{S(t)},$$

where the fraction represents the conditional probability of surviving the next ten years given that the patient has survived to age t. Similarly, the conditional 1-year risk estimate $R_1(t)$ can be written as $R_1(t) = 1 - S(t+1)/S(t)$. The fraction can be expanded as follows:

$$\begin{aligned}
R_1(t) &= 1 - \frac{S(t+1)}{S(t)} \\
&= 1 - \frac{S(t+1)}{S(t)} \times \frac{S(t-10)}{S(t-10)} \times \frac{S(t-9)}{S(t-9)} \\
&= 1 - \frac{S(t+1)}{S(t-9)} \times \frac{S(t-10)}{S(t)} \times \frac{S(t-9)}{S(t-10)} \\
&= 1 - \big(1 - R_{10}(t-9)\big) \times \frac{1}{1 - R_{10}(t-10)} \times \big(1 - R_1(t-10)\big). \qquad (1)
\end{aligned}$$

For a 30-year-old patient, the 1-year risk is $1 - S(31)/S(21) \times S(20)/S(30) \times S(21)/S(20)$, where the first two fractions can be obtained from the 10-year risk estimate $R_{10}(t)$ (e.g., $S(31)/S(21) = 1 - R_{10}(21)$). Assume that the 1-year risk for patients aged 20-29 years is 0 % (i.e., $R_1(t) = 0$ for all $t \in \{20{,}29\}$). This is not a strong assumption since the 10-year risk of these age groups based on FINRISK function lies between 0.2 % and 0.4 %. Now, the conditional 1-year survival probabilities as well as risk estimates of patients aged 30 years or more can be calculated through (1).

Based on these 1-year risk estimates and the assumption that an average CHD event occurs in the middle of the year given that it occurs during that particular year, the expected timing of a CHD event of a patient aged t years can be calculated as follows:

$$E[\text{CHD timing}](t) = \sum_{i=0}^{9} \left[(i - 0.5) \frac{R_1(t+i)}{\sum_{j=0}^{9} R_1(t+j)} \right].$$

The average timing of a CHD event in the population is obtained as a weighted average of $E[\text{CHD timing}](t)$, where the weights reflect the European standard population [9]. For patient aged 45+ years, the average timing of a CHD event is 5.75 years after the risk evaluation, given that the event occurs in the 10-year period.

References

1. Ripatti S, Tikkanen E, Orho-Melander M, Havulinna AS, Silander K, Sharma A, et al. A multilocus genetic risk score for coronary heart disease: case-control and prospective cohort analyses. Lancet. 2010;376(9750):1393–400.
2. Tikkanen E, Havulinna AS, Palotie A, Salomaa V, Ripatti S. Genetic risk prediction and a 2-stage risk screening strategy for coronary heart disease. Arterioscler Thromb Vasc Biol. 2013;33(9):2261–6.
3. Vartiainen E, Laatikainen T, Salomaa V, Jousilahti P, Peltonen M, Puska P. Sydäninfarkti- ja aivohalvausriskin arviointi FINRISKI-tutkimuksessa (The FINRISK FUNCTION: Estimation of the risk of coronary events and stroke in the Finnish population). Suom Lääkäril. 2007;48(5):4507–13.
4. Anderson KM, Odell PM, Wilson PWF, Kannel WB. Cardiovascular disease risk profiles. Am Heart J. 1991;121(1):293–8.
5. Wilson PWF, D'Agostino RB, Levy D, Belanger AM, Silbershatz H, Kannel WB. Prediction of coronary heart disease using risk factor categories. Circulation. 1998;97(18):1837–47.
6. Abraham G, Havulinna AS, Bhalala OG, Byars SG, De Livera AM, Yetukuri L, et al. Genomic prediction of coronary heart disease. Eur Heart J. 2016;37(43):3267–78.
7. Statistics Finland. Population Structure. Official Statistics of Finland. 2017 [cited 2017 Dec 1]. Available from: http://www.stat.fi/til/vaerak/index_en.html
8. Borodulin K, Levälahti E, Saarikoski L, Lund L. Kansallinen FINRISKI 2012-terveystutkimus-Osa 2: Tutkimuksen taulukkoliite. Raportti: 2013 [cited 2016 Jul 1]. Available from: http://julkari.fi/handle/10024/114942
9. Pace M, Lanzieri G, Glickman M, Grande E, Zupanic T, Wojtyniak B, et al. Revision of the European Standard Population: report of Eurostat's task force. 2013.
